# N-Acetylglucosamine Kinase–Small Nuclear Ribonucleoprotein Polypeptide N Interaction Promotes Axodendritic Branching in Neurons via Dynein-Mediated Microtubule Transport

**DOI:** 10.3390/ijms241411672

**Published:** 2023-07-19

**Authors:** Binod Timalsina, Ho Jin Choi, Il Soo Moon

**Affiliations:** Department of Anatomy, Dongguk University College of Medicine, Gyeongju 38066, Republic of Korea; binodtimalsina19@gmail.com (B.T.); chjack@naver.com (H.J.C.)

**Keywords:** microtubule transport, NAGK, neuronal complexity, Prader–Willi syndrome, SNRPN

## Abstract

N-acetylglucosamine kinase (NAGK) has been identified as an anchor protein that facilitates neurodevelopment with its non-canonical structural role. Similarly, small nuclear ribonucleoprotein polypeptide N (SNRPN) regulates neurodevelopment and cognitive ability. In our previous study, we revealed the interaction between NAGK and SNRPN in the neuron. However, the precise role in neurodevelopment is elusive. In this study, we investigate the role of NAGK and SNRPN in the axodendritic development of neurons. NAGK and SNRPN interaction is significantly increased in neurons at the crucial stages of neurodevelopment. Furthermore, overexpression of the NAGK and SNRPN proteins increases axodendritic branching and neuronal complexity, whereas the knockdown inhibits neurodevelopment. We also observe the interaction of NAGK and SNRPN with the dynein light-chain roadblock type 1 (DYNLRB1) protein variably during neurodevelopment, revealing the microtubule-associated delivery of the complex. Interestingly, NAGK and SNRPN proteins rescued impaired axodendritic development in an SNRPN depletion model of Prader–Willi syndrome (PWS) patient-derived induced pluripotent stem cell neurons. Taken together, these findings are crucial in developing therapeutic approaches for neurodegenerative diseases.

## 1. Introduction

Neurodevelopment is a complex process that involves the interaction of various genes and proteins. N-acetylglucosamine kinase (NAGK) is an enzyme that phosphorylates N-acetylglucosamine (GlcNAc) to produce GlcNAc-6-phosphate (GlcNAc-6-P), which is further utilized to synthesize oligosaccharide chains, glycolipids [1,2], and glycosaminoglycan [3]. The interacting partner, the small nuclear ribonucleoprotein polypeptide N (*SNRPN*) gene located on the 15q11-q13 region of the human chromosome, encodes for the SmN protein and is also associated with Prader–Willi syndrome/Angelman syndrome (PWS/AS) [4]. In our previous study, NAGK and SNRPN interaction in the dendrites of developing neurons was reported [5]. However, the precise interaction and the involvement of NAGK and SNRPN in neurodevelopment are not well understood. The non-canonical role of the NAGK protein in neuronal development has been explored [6,7]. SNRPN, on the other hand, is a protein that is primarily expressed in the brain and is involved in the regulation of alternative splicing of pre-mRNA [8,9,10]. NAGK is highly expressed in the neurons of the cerebral cortex and hippocampus, whereas the expression is reduced in astrocytes and oligodendrocytes [11]. The expression of SNRPN is significantly higher in the cerebral cortex and hippocampus as compared to the cerebellum [12]. In this study, we revealed the normal expression of NAGK and SNRPN in a developing neuron. The involvement of SNRPN and NAGK interaction in neuronal dendrites indicates their involvement in the local splicing process [5]. Through using a combination of molecular biology and imaging techniques, we gained a better understanding of the mechanisms underlying NAGK–SNRPN interaction and its impact on neuronal development and function.

Reports also suggested the crucial role of SNRPN in the regulation of neuronal-specific alternative splicing events [13]. Since neurodevelopment is a complex process that involves the precise regulation of gene expression and protein activity [14], the involvement of NAGK and SNRPN interaction can be important in neuronal development and function. Previous studies have reported NAGK distribution throughout neurons until the axonal outgrowth stage and its reduction during dendritic outgrowth to a negligible amount in the mature stage [6]. Moreover, NAGK overexpression upregulated dendritic arborization, and vice versa, with the knockdown of the NAGK protein [7,11]. However, the action of NAGK in different stages of neurodevelopment was unidentified. Similarly, the role of SNRPN in neurite outgrowth, dendritic spine formation, and neuronal migration was reported [12]. In addition, SNRPN was also identified as a potential factor regulating axonal regeneration in an animal study [15]. Although NAGK and SNRPN interaction was identified in the neuron, their combined action on neurodevelopment is not disclosed yet. We investigated the effect of overexpression and knockdown of NAGK and SNRPN proteins in the early and late stages of neurodevelopment. Additionally, we also explored the potential implications of disrupting NAGK and SNRPN interaction in neurodevelopment and its association with neurological disorders.

NAGK also interacts with the dynein light-chain roadblock type 1 (DYNLRB1) protein in developing neurons [16]. However, the potential role of dynein-mediated transport of the two proteins, NAGK and SNRPN, in neurodevelopment is still unclear. The direct interaction of NAGK with DYNLRB1 verified the structural role of NAGK in microtubule transport in neurons [16]. Additionally, neural trafficking of the SmB protein was reported previously [17]. Despite these findings, the precise mechanisms through which NAGK and SNRPN contribute to neurodevelopment remain elusive. The dynein complex is a large structure made up of six distinct subunits such as heavy chains, intermediate chains, light intermediate chains, and three types of light chains with the DYNLRB1 as one of them [18]. These motors play a critical role in the transportation and distribution of organelles, signaling complexes, and cytoskeletal components [19]. Dynein-powered retrograde transport of organelles and proteins from the axon to the cell body is crucial in neurobiology, and disrupted transport may contribute to neurodegenerative conditions [20,21,22]. Ciliobrevins are widely used to block dynein-dependent microtubule transport in cells [23]. Moreover, microtubule-mediated transport can be assessed using drugs like nocodazole, which modifies microtubule polymerization [24]. To elucidate dynein-mediated microtubule transport, we used a melanoma cell line to observe the colocalization of NAGK or SNRPN protein transport with melanosome.

PWS is a genetic disorder characterized by intellectual disability, hyperphagia, hypotonia, and other developmental abnormalities [25]. The paternally expressed 15q11-q13 region of chromosome 15 encodes five polypeptide coding genes such as bicistronic *SNURF-SNRPN*, *MKRN3*, *MAGEL2*, and *NECDIN* [26]. The 15q11-q13 region l expresses the paternal SNRPN gene and its downstream noncoding region [27,28]. The involvement of SNRPN in RNA splicing and the post-translational modification of protein is widely accepted; however, its role in neurodevelopment is yet to be disclosed [4]. The use of induced pluripotent stem cell (iPSC) technology is a highly valuable method for generating in vitro models of human genetic disorders, especially in cases where suitable animal models are lacking or species comparison is challenging, such as with imprinting diseases like PWS, a neurogenetic disorder [29,30]. The role of NAGK and SNRPN in the PWS patient’s brain is less understood. In this study, we explored the role of NAGK and SNRPN on an SNRPN-depleted PWS patient iPSC-derived neuron. Overexpression and knockdown of the NAGK protein along with the SNRPN overexpression rescued axodendritic development, which provides some clues to derive possible therapeutic approaches in genetic neurodegenerative diseases such as PWS. 

Our findings indicate that the dynein-mediated microtubule transport of the NAGK–SNRPN complex is involved in the axodendritic branching of the neuron.

## 2. Results

### 2.1. NAGK and SNRPN Interact Variably in the Cell Body and Processes of Primary Neurons during Neurodevelopment

**Figure 1:**
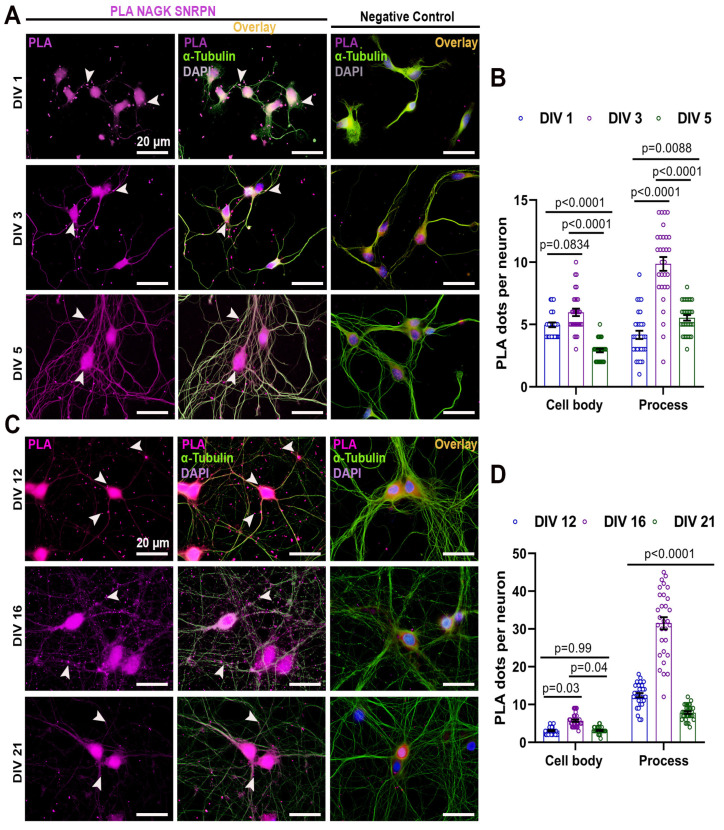
Proximity ligation assay (PLA) showing normal expression of NAGK and SNRPN in primary neurons. (**A**) NAGK–SNRPN PLA dots (magenta pointed with arrowheads) were observed at DIV 1/3/5 and negative control. Immunocytochemistry (ICC) with α-tubulin (green) was used to observe neuron morphology. (**B**) Bar graphs showing mean NAGK–SNRPN PLA dots at the cell body and process in primary hippocampal neuron from DIV 1/3/5. (**C**) NAGK–SNRPN PLA dots were observed at DIV 12/16/21. α-Tubulin (green) was used to stain for neuron morphology. (**D**) Bar graphs showing mean NAGK–SNRPN PLA dots at the cell body and process in primary hippocampal neuron from DIV 12/16/21. Bars represent the mean ± SEM (*n* = 30 neurons) PLA dots. Statistical significance was compared using two-way ANOVA with Sidak’s multiple comparisons tests: *p* < 0.05, *p* < 0.01, and *p* < 0.001. Scale bar of 20 µm applies to all the images.

At first, we wondered about the distribution of NAGK–SNRPN interaction at the different stages of neurodevelopment. We performed a proximity ligation assay (PLA) with antibodies specific to the NAGK and SNRPN proteins to observe NAGK and SNRPN interaction in primary hippocampal culture categorized as early (DIV 1/3/5) and late stages (DIV 12/16/21) (Figure 1). The neuron morphology was observed via ICC using an α-tubulin antibody (green) and nuclear stain (DAPI) to visualize the cell body. Negative control PLA with the single primary antibody was performed with the corresponding DIV neuron (Figure 1A–C). The PLA dots (magenta) and α-tubulin ICC were colocalized as the overlay. At the early stages of development, DIV 3 neurons exhibited maximal PLA dots in the cell body (*p* < 0.001) (Figure 1A,B), and the PLA dots were significantly increased (*p* < 0.001) from DIV 1–5 in neuronal processes (Figure 1A,B). Similarly, PLA was performed at the late stages of neurodevelopment (Figure 1C). The NAGK–SNRPN PLA dots were significantly higher in the cell body at DIV 16 (*p* < 0.001) and reduced significantly at DIV 21 (*p* < 0.001) (Figure 1C,D). However, the PLA dots reduced gradually from DIV 12–21 in the neuronal process (Figure 1C,D).

To further validate NAGK–SNRPN interaction, we performed a Western blot (WB) assay from the protein lysates collected during the different developmental stages of the neuron, i.e., the early (in vitro, DIV 1/3/5 and in vivo P1/3/5) and late (in vitro, DIV 12/16/21 and in vivo P12/16/21) phases. The protein lysate was collected from the primary hippocampal culture at DIV 1/3/5/12/16/21 as the mouse pup’s brains from P1/3/5/12/16/21. NAGK and SNRPN expression at different stages of neurodevelopment was confirmed via the WB analysis (Figure S1). Expression of NAGK and SNRPN was significantly increased at DIV 3/P3 at the early stages of neurodevelopment in primary neurons (*p* < 0.001) (Figure S1A,B) and mouse pups (*p* < 0.05) (Figure S1A,C). In the late phases of neurodevelopment, NAGK and SNRPN expression was significantly increased (*p* < 0.001) at DIV 12–16/P12–16 compared to the DIV 21/P 21 lysates (Figure S1D,F).

### 2.2. NAGK Interacts Directly with SNRPN in the In Vitro Binding Assay

**Figure 2:**
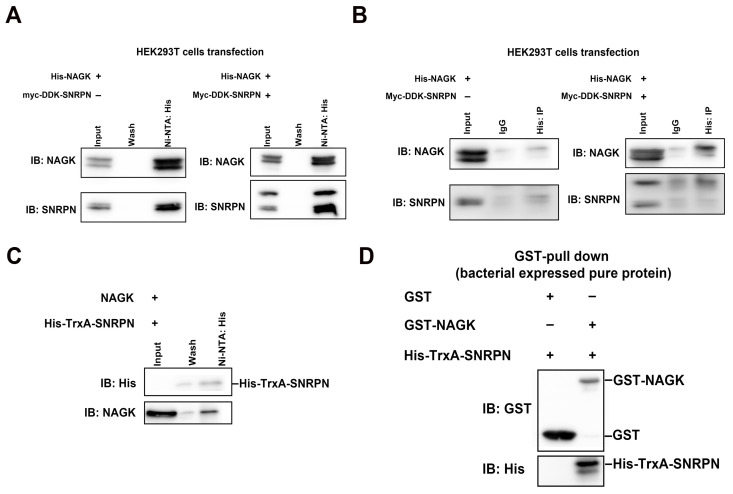
Pull-down and co-immunoprecipitation assays showing NAGK and SNRPN interaction. (**A**) His pull-down assay showing the interaction with His-NAGK and Myc-DDK-SNRPN in HEK293T cell lysate. (**B**) Co-immunoprecipitation assay showing the interaction between His-NAGK and Myc-DDK-SNRPN in HEK293T cell lysate. (**C**) His pull-down assay showing the interaction between HEK293T-cell-expressed NAGK and bacterial-expressed purified His-TrxA-SNRPN. (**D**) GST pull-down assay showing the direct interaction between GST-NAGK and His-TrxA-SNRPN purified proteins expressed in bacteria.

Although NAGK and SNRPN interaction was verified via yeast-two hybrid assay previously [5], in vitro binding assay data is lacking. So, we validated our previous finding via in vitro binding assay to confirm the direct interaction between NAGK and SNRPN proteins. We performed His/Glutathione S-transferase (GST) pull-down and co-immunoprecipitation assays (Figure 2). The his-tagged NAGK plasmid was co-transfected with or without Myc-DDK-SNRPN plasmids in HEK293T cells for 48 h. An equal amount of protein lysate (500 µg) was pulled down using the Ni-NTA-based technique. His-NAGK protein pulled the endogenous and exogenous SNRPN (Figure 2). Similarly, the Ni-NTA-based technique was cross-checked using a co-immunoprecipitation (Co-IP) assay using the His primary antibody (4 µg) and the control IgG (4 µg) in Protein G agarose beads. Here also, His-NAGK pulled the endogenous and exogenous SNRPN (Figure 2B). Additionally, NAGK–SNRPN interaction was confirmed via a His-pull down assay with the bacterial-expressed pure protein His-TrxA-SNRPN and mammalian-expressed NAGK (Figure 2C). Finally, the direct interaction of NAGK and SNRPN was confirmed using a GST-pull down assay. The GST control and GST-NAGK expressed in bacteria were purified and a GST-pull down assay was performed with the pure His-TrxA-SNRPN protein expressed in bacteria (Figure 2D).

### 2.3. Overexpression and Knockdown of NAGK and SNRPN Promote Axodendritic Outgrowth in the Early and Late Stages of Neurodevelopment

**Figure 3:**
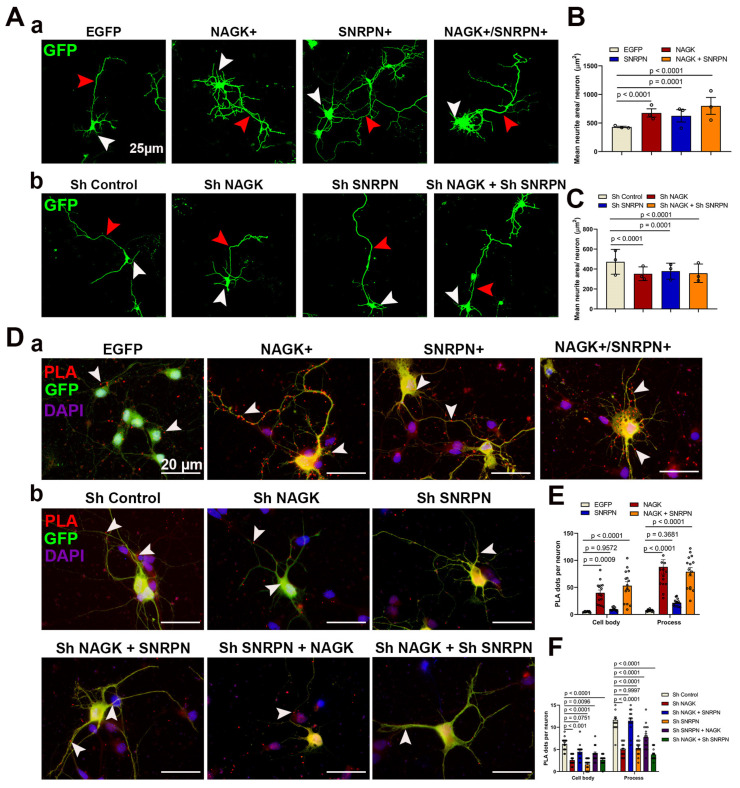
Overexpression and knockdown effect of NAGK and SNRPN in early stages (DIV 4) of neurodevelopment. (**A**): (**A-a**) Neuron morphology showing the overexpression effect of NAGK and SNRPN (GFP, green). (**A-b**) Neuron morphology showing the knockdown effect of NAGK and SNRPN (GFP, green). (**B**) Bar graphs showing the mean neurite area with NAGK and SNRPN overexpression. (**C**) Bar graphs showing the mean neurite area with NAGK and SNRPN knockdown. (**D**): (**D-a**) NAGK–SNRPN PLA (red) with NAGK and SNRPN overexpression with neuron morphology in GFP (green) and cell body (DAPI). (**D-b**) NAGK–SNRPN PLA (red) with NAGK and SNRPN knockdown neurons (GFP, green), cell body (DAPI). (**E**) Bar graphs showing the mean PLA dots with NAGK and SNRPN overexpression. (**F**) Bar graphs showing mean PLA dots with NAGK and SNRPN knockdown. Bars represent the mean ± SEM (*n* = 15 neurons) PLA dots. Statistical significance was evaluated using one-way ANOVA with Tukey’s multiple comparisons test, denoted as *p* < 0.05, *p* < 0.01, and *p* < 0.001. A scale bar of 20 µm applies to all the images. Arrowheads red and white showed the neuronal process and PLA dots.

To confirm the action of NAGK and SNRPN on neurons, we observed the overexpression and knockdown effects of NAGK and SNRPN in the early stages of neurodevelopment in primary hippocampal culture at DIV 4 (Figure 3A–C). The neurons were transfected at DIV 2 with recombinant adeno-associated viral (AAV) particles carrying NAGK and SNRPN plasmids along with the reporter EGFP plasmid. The morphology of the transfected neurons was studied at DIV 4, 48 h after transgene introduction. The overexpression of NAGK and SNRPN with co-transfection of NAGK–SNRPN significantly increased (*p* < 0.001) the neurite mean area calculated via the NeurphologyJ plugins in Image J software Version 1.43 (Figure 3A-a,B) [31]. We also observed the knockdown effect of NAGK and SNRPN plasmids along with the control co-transfected with the reporter EGFP plasmid (Figure 3A-b). The neurite area was significantly reduced (*p* < 0.001) through the knockdown of the NAGK and SNRPN proteins as compared to the control plasmid (Figure 3C). Furthermore, we validated the overexpression and knockdown effect of NAGK and SNRPN via PLA (Figure 3D-a,b). The mean NAGK–SNRPN PLA dots were significantly increased (*p* < 0.001) in the cell body (DAPI) and the process (GFP) of the neurons with NAGK, SNRPN, and NAGK + SNRPN plasmid overexpression in the neuron (Figure 3E), whereas a significant decrement (*p* < 0.001) of the mean NAGK–SNRPN PLA dots was observed with NAGK, SNRPN, and NAGK + SNRPN protein knockdown in the cell body and processes of DIV 4 neurons (Figure 3F).

**Figure 4:**
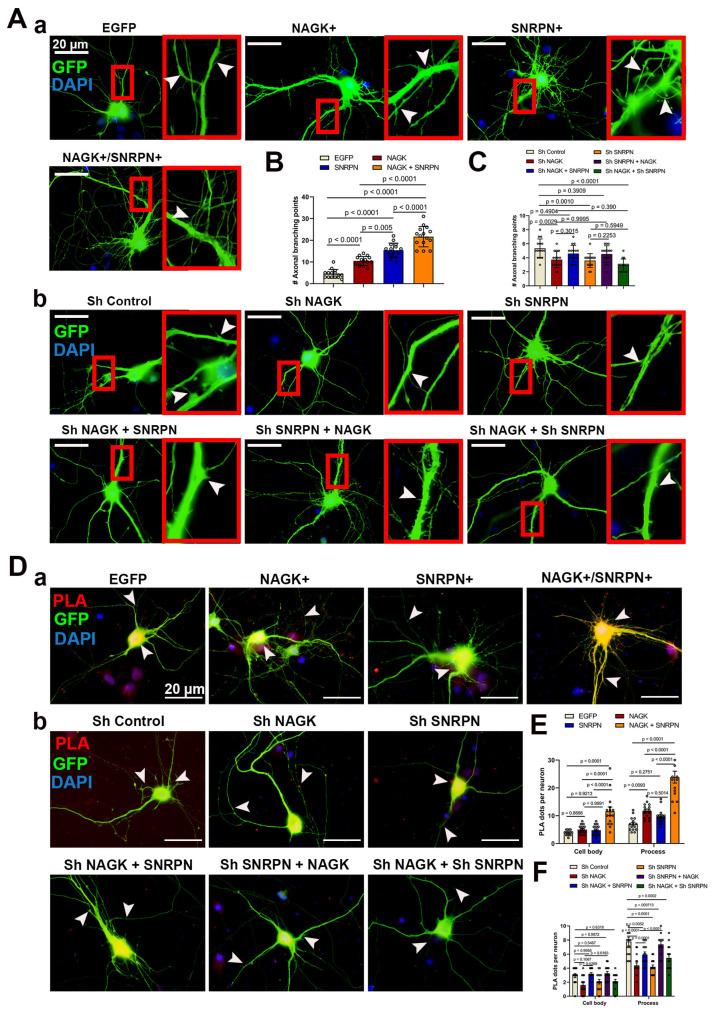
Overexpression and knockdown of NAGK and SNRPN in late stages of neurodevelopment (DIV 14). (**A**) (**A**-**a**) Neuron morphology showing the overexpression effect of NAGK and SNRPN. (**A**-**b**) Neuron morphology showing the knockdown effect of NAGK and SNRPN. (**B**) Bar graphs showing the mean axonal branching points with NAGK and SNRPN overexpression. (**C**) Bar graphs showing the mean axonal branching points with NAGK and SNRPN knockdown. (**D**) (**D**-**a**) NAGK–SNRPN PLA with NAGK and SNRPN overexpression. (**D**-**b**) NAGK–SNRPN PLA with NAGK and SNRPN knockdown. (**E**) Bar graphs showing the mean PLA dots with NAGK and SNRPN overexpression. (**F**) Bar graphs showing the mean PLA dots with NAGK and SNRPN knockdown. The bars represent the mean ± SEM (*n* = 15 neurons) of PLA dots. Statistical significance was assessed using one-way ANOVA with Tukey’s multiple comparisons tests, indicated as *p* < 0.05, *p* < 0.01, and *p* < 0.001. A scale bar of 20 µm applies to all the images, with an inset (red box) scale bar of 5.8 µm. Arrowheads (white) showed the neuronal process and PLA dots.

Similar to the early-stage experiment, we observed the effects of NAGK and SNRPN overexpression and knockdown in the late stages of neurodevelopment in primary hippocampal cultures at DIV 14 (Figure 4A–C). Neurons were transfected with NAGK and SNRPN plasmids along with the EGFP plasmid at DIV 12, and the morphology of transfected neurons was analyzed at DIV 14, 48 h after transgene introduction. Overexpression of NAGK and SNRPN, as well as co-transfection of NAGK–SNRPN, significantly increased (*p* < 0.001) the frequency of axodendritic branching (Figure 4A-a,B). Additionally, knockdown of NAGK and SNRPN using specific plasmids, along with a control plasmid co-transfected with EGFP, significantly reduced (*p* < 0.001) axodendritic branching compared to the control plasmid (Figure 4A-b,C). We also further validated the overexpression and knockdown effects of NAGK and SNRPN via PLA using NAGK and SNRPN primary antibodies in transfected neurons (Figure 4D-a,b). The mean number of NAGK–SNRPN PLA dots was significantly increased (*p* < 0.001) in the cell body and processes of neurons with NAGK, SNRPN, and NAGK + SNRPN overexpression (Figure 4E). Conversely, a significant decrease (*p* < 0.001) in the mean number of NAGK–SNRPN PLA dots was observed with NAGK, SNRPN, and NAGK + SNRPN knockdown in the cell body and processes of DIV 14 neurons (Figure 4F).

### 2.4. NAGK and SNRPN Interact Variably with DYNLRB1 during Neurodevelopment

**Figure 5:**
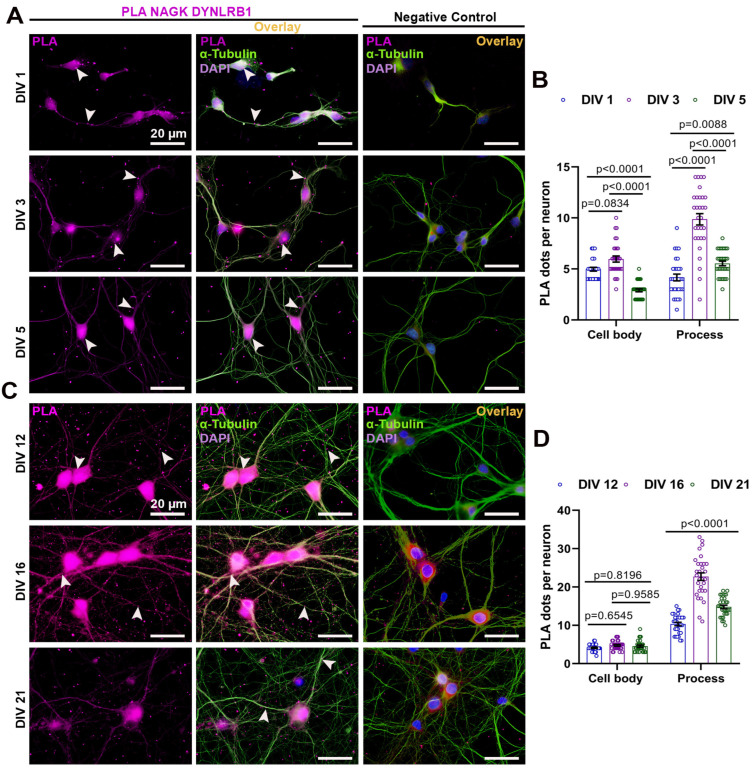
Proximity ligation assay (PLA) showing normal expression of NAGK and DYNLRB1 in primary neurons. (**A**) NAGK–DYNLRB1 PLA dots (magenta shown with arrowheads) were observed at DIV 1/3/5 and negative control. ICC with α-tubulin (green) was used to observe neuron morphology. (**B**) Bar graphs showing the mean NAGK–DYNLRB1 PLA dots at the cell body and process in primary hippocampal neuron from DIV 1/3/5. (**C**) NAGK–DYNLRB1 PLA dots were observed at DIV 12/16/21. ICC for α-tubulin (green) was used to stain the neuron morphology. (**D**) Bar graphs showing the mean NAGK–DYNLRB1 PLA dots at the cell body and process in primary hippocampal neuron from DIV 12/16/21. Bars represent the mean ± SEM (*n* = 30 neurons) PLA dots. Statistical significance was assessed using two-way ANOVA with Sidak’s (DIV 1–5) and Tukey’s (DIV 12–21) multiple comparisons tests, with *p* < 0.05, *p* < 0.01, and *p* < 0.001 indicating significance levels. Scale bar of 20 µm applies to all the images.

To visualize direct protein interaction, PLA is limited to two host antibodies. So, we chose to perform the NAGK–DYNLRB1 and SNRPN–DYNLRB1 PLAs separately to disclose the specific interaction during neurodevelopment. At first, we studied the interaction of NAGK and DYNLRB1 proteins in primary hippocampal cultures at different developmental stages (Figure 5). Neuronal morphology was visualized using an α-tubulin antibody (green) and nuclear stain (DAPI) via ICC to observe the cell body. A negative control PLA, with only a single primary antibody, was performed on corresponding DIV neurons (Figure 5A–C). The PLA dots (magenta), representing NAGK–DYNLRB1 interaction, were colocalized with α-tubulin ICC overlay. The maximal PLA dots were observed in the cell body of DIV 1/3 neurons (*p* < 0.001) at the early stages of development (Figure 5A,B), and the PLA dots in neuronal processes also significantly increased (*p* < 0.001) at DIV 3 (Figure 5B). Similarly, PLA was performed at the late stages of neurodevelopment (Figure 5C), where NAGK–DYNLRB1 PLA dots were significantly higher in the cell body and process of the neuron at DIV 16 (*p* < 0.001) and reduced significantly at DIV 21 (*p* < 0.001) (Figure 5C,D).

**Figure 6:**
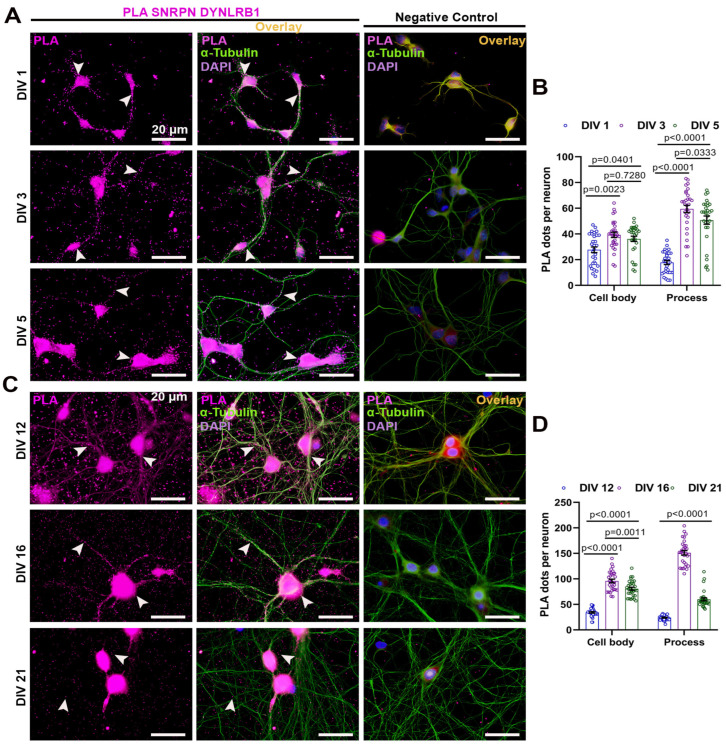
Proximity ligation assay showing normal expression of SNRPN and DYNLRB1 in primary neurons. (**A**) SNRPN–DYNLRB1 PLA dots (magenta shown with arrowheads) were observed at DIV 1/3/5 and negative control. ICC with α-tubulin (green) was used to stain the neuron morphology. (**B**) Bar graphs showing the mean SNRPN–DYNLRB1 PLA dots at the cell body and processes in primary hippocampal neurons from DIV 1/3/5. (**C**) SNRPN–DYNLRB1 PLA dots were observed at DIV 12/16/21. ICC with α-tubulin (green) was used to visualize neuron morphology. (**D**) Bar graphs showing the mean NAGK–SNRPN PLA dots at the cell body and process in a primary hippocampal neuron from DIV 12/16/21. Bars indicate mean ± SEM (*n* = 30 neurons) PLA dots. Statistical significance was determined using two-way ANOVA with Tukey’s multiple comparisons tests: *p* < 0.05, *p* < 0.01, and *p* < 0.001. Scale bar of 20 µm applies to all images.

Similarly, we observed the interaction between SNRPN and DYNLRB1 proteins in primary hippocampal cultures at different developmental stages (Figure 6). Neuronal morphology was visualized through staining with α-tubulin antibody (green) and nuclear stain (DAPI) using ICC to observe the cell body. A negative control PLA, involving only a single primary antibody, was performed on corresponding DIV neurons, as shown in Figure 6A,C. The colocalization of PLA dots, representing SNRPN–DYNLRB1 interaction (magenta), with α-tubulin (green) ICC overlay was observed in the neuron at the early and late neurodevelopmental stages. The highest number of PLA dots was observed in the cell body of DIV 3 neurons (*p* < 0.001) during the early stages of development, as shown in Figure 6A,B, and the PLA dots in neuronal processes also significantly increased (*p* < 0.001) at DIV 3, as shown in Figure 6B. Similarly, PLA was performed during the late stages of neurodevelopment, as shown in Figure 6C, and the SNRPN–DYNLRB1 PLA dots were significantly higher in the cell body and processes of the neuron at DIV 16 (*p* < 0.001), but reduced significantly at DIV 12 and 21 (*p* < 0.001), as shown in Figure 6C,D.

### 2.5. DYNLRB1 Interacts with NAGK and SNRPN in the In Vitro Binding Assay

To confirm the direct interaction between NAGK and SNRPN proteins with DYNLRB1, we conducted an in vitro binding assay using the His pull-down assay, as shown in Figure S2. NAGK and DYNLRB1 interaction was previously reported from our lab [16,32]. HEK293T cells were co-transfected with His-tagged NAGK, His-DYNLRB1, Myc-DDK-SNRPN, and Myc-DDK-DYNLRB1 plasmids with alternate pairing of His and DDK tags for the His pull-down assay. Protein lysates were collected after 48 h of transfection and equal amounts (500 µg) were subjected to pull-down using the Ni-NTA-based technique. His-NAGK protein pulled down both endogenous and exogenous DYNLRB1 proteins, as demonstrated in the Western blot images (Figure S2A). Further, in another set of experiments, His-DYNLRB1 and Myc-DDK SNRPN plasmids were co-transfected or a His-DYNLRB1 plasmid was transfected alone in HEK293T cells, and His pull-down was conducted in an equal amount (500 µg) of proteins. His-DYNLRB1 interacted with both the endogenous and exogenous SNRPN protein in the Ni-NTA assay, as shown in Figure S2B.

### 2.6. NAGK, SNRPN, and DYNLRB1 Colocalized on the Microtubules of SK-MEL-31 Cell Lines

To describe NAGK–SNRPN complex variability in the cell body and process of the neuron, we observed the microtubule transport machinery of NAGK and SNRPN in a melanoma cell line model. We performed ICC to observe the colocalization between NAGK, SNRPN, and DYNLRB1 on microtubules using SK-MEL-31 cells. The NAGK proteins were colocalized on the microtubule (Figure S3A), which has been reported from our lab previously [6]. Also, we observed the colocalization of SNRPN protein on the microtubules (Figure S3B). In Figure S3C, NAGK (red) and SNRPN (green) colocalization (overlay) were observed on the microtubules throughout the cell morphology. We also demonstrated NAGK or SNRPN (red) and DYNLRB1 (green) colocalization (overlay) along the microtubules (Figure S3D,E).

### 2.7. NAGK, SNRPN, and DYNLRB1 Group on the Microtubules with Dynein Inhibition and Disperse during Microtubule Disruption

**Figure 7:**
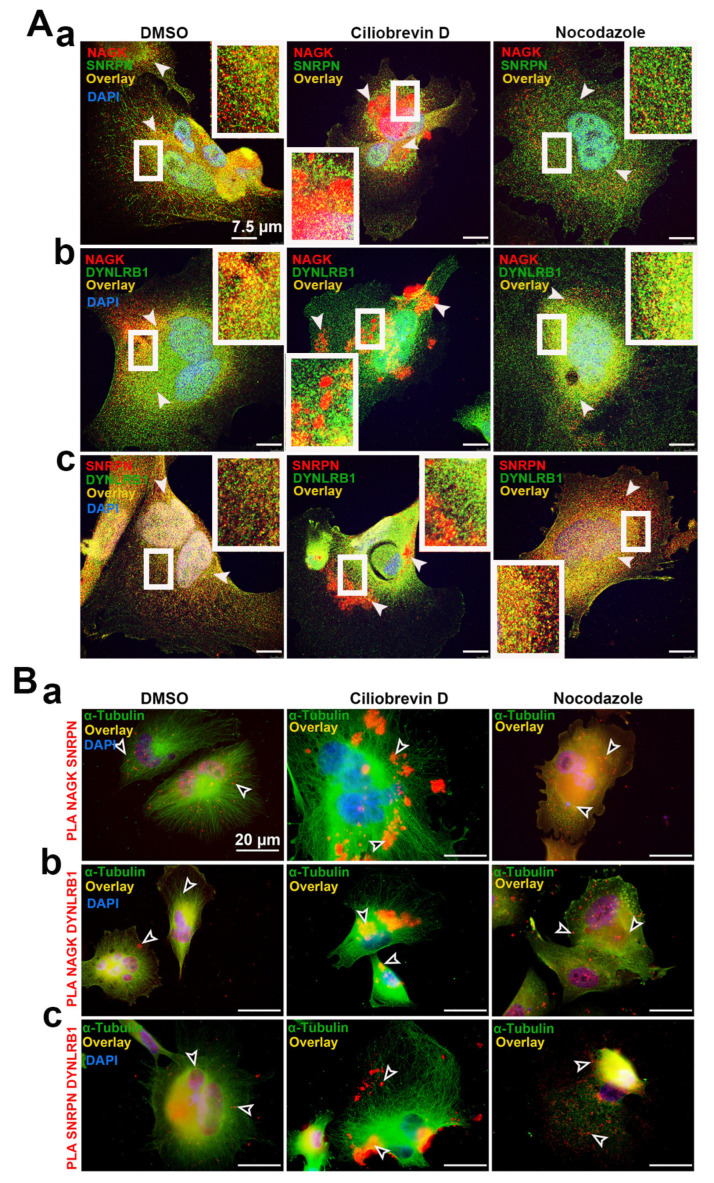
Immunocytochemistry and proximity ligation assay showing NAGK, SNRPN, and DYNLRB1 colocalization on microtubules with ciliobrevin D and nocodazole treatments in SK-MEL-31 cells. (**A**) ICC showing the treatments with DMSO, ciliobrevin D, and nocodazole: (**A**-**a**) NAGK (red), SNRPN (green), and colocalization (overlay shown with arrowheads); (**A**-**b**) NAGK (red), DYNLRB1 (green), and colocalization (overlay); (**A**-**c**) SNRPN (red), DYNLRB1 (green), and colocalization (overlay). (**B**) PLA showing treatments with DMSO, ciliobrevin D, and nocodazole: (**B**-**a**) NAGK –SNRPN; (**B**-**b**) NAGK–DYNLRB1; (**B**-**c**) SNRPN–DYNLRB1 and colocalization (overlay shown with white arrowheads). ICC with α-tubulin demonstrating microtubule distribution. Scale bar, 7.5 µm (**A**) and 20 µm (**B**) applies to all the images. White box inset showed the magnified colocalization in overlay.

We further confirmed the dynein-mediated transport of the NAGK–SNRPN complex via two techniques in the SK-MEL-31 cells: (a) blocking dynein movement using the dynein inhibitor (ciliobrevin D) and (b) disrupting the microtubule using nocodazole. The microtubule transport mechanism of NAGK and SNRPN was observed using the dynein inhibitor (ciliobrevin D, 80 µM for 6 h) and microtubule-disrupting compound nocodazole (10 µM for 1 h) in SK-MEL-31 cells (Figure 7). The control cells were treated with vehicle (DMSO). ICC was performed to observe the NAGK (red) and SNRPN (green) colocalization (overlay) with the treatments DMSO, ciliobrevin D, and nocodazole, respectively (Figure 7A-a). The distributed NAGK–SNRPN puncta appeared to be accumulated on the microtubules, making patches during ciliobrevin D treatment. However, dispersed colocalized puncta as appeared in the DMSO treatment were observed in the nocodazole treatment following microtubule disruption. We also observed a similar phenomenon with NAGK (red)–DYNLRB1 (green) (Figure 7A-b) and SNRPN (red)–DYNLRB1 (green) (Figure 7A-c) ICC in SK-MEL-31 cells. Further validation of the ICC experiment was performed via PLA in a similar experiment setup in SK-MEL-31 cells (Figure 7B). NAGK–SNRPN PLA (red) dots were grouped on the microtubules after dynein inhibition, whereas scattered PLA dots were observed in microtubule breakdown, as shown in Figure 7B-a. NAGK–DYNLRB1 and SNRPN–DYNLRB1 PLA dots were also grouped as puncta over the microtubules during the ciliobrevin D treatment (Figure 7B-b,c).

### 2.8. Melanosomes Colocalize with NAGK and SNRPN during Dynein Inhibition and Microtubule Disruption

We attempted to demonstrate melanosome colocalization with the NAGK and SNRPN proteins since we used the melanosome trafficking pathway as a model to study NAGK, SNRPN, and DYNLRB1 interaction. We analyzed the colocalization of the NAGK and SNRPN proteins via ICC in the melanosome trafficking process with the melanosome-specific primary antibody (PMEL). Melanosomes were treated with vehicle (DMSO), ciliobrevin D (80 µM for 6 h), and nocodazole (10 µM for 1 h). ICC was performed with NAGK (red) and PMEL (green) primary antibodies and colocalization (overlay) was observed on the SK-MEL-31 cells (Figure S4A). Similarly, SNRPN (red) and PMEL (green) primary antibodies were used for ICC, and colocalization (overlay) was observed on the SK-MEL-31 cells (Figure S4B). The NAGK and SNRPN protein puncta observed during ciliobrevin D treatment were colocalized with the melanosomes, while nocodazole treatment resulted in scattered colocalized NAGK–PMEL and SNRPN–PMEL puncta via microtubule disruption.

### 2.9. Melanosome Distribution Varied during Overexpression and Knockdown of NAGK, SNRPN, and DYNLRB1 Proteins with Dynein Inhibition and Microtubule Disruption

**Figure 8:**
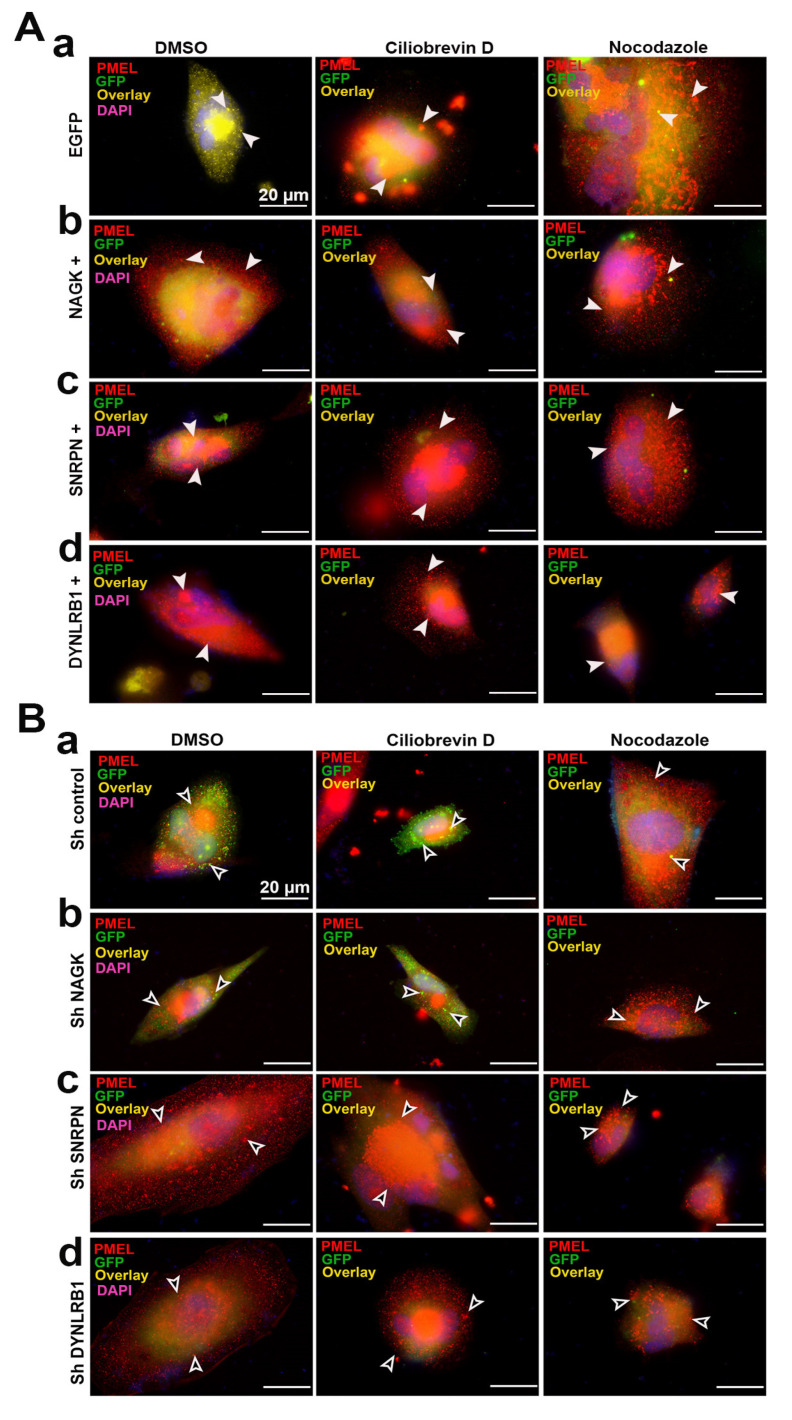
Immunocytochemistry showing NAGK, SNRPN, and DYNLRB1 colocalization with melanosome in SK-MEL-31 cell line. (**A**) ICC showing the treatments with (DMSO, ciliobrevin D, and nocodazole) PMEL (red), GFP (green), and colocalization (overlay shown with arrowheads); (**A**-**a**) EGFP, (**A**-**b**) NAGK, (**A**-**c**) SNRPN, and (**A**-**d**) DYNLRB1 were overexpressed in the SK-MEL-31 cell line co-transfected with EGFP. (**B**) ICC showing the treatments with (DMSO, ciliobrevin D, and nocodazole) PMEL (red), GFP (green), and colocalization (overlay); (**B**-**a**) EGFP, (**B**-**b**) NAGK, (**B**-**c**) SNRPN, and (**B**-**d**) DYNLRB1 were knocked down in the SK-MEL-31 cell line co-transfected with EGFP. Scale bar of 20 µm applies to all the images. Scale bar of 20 µm applies to all the images.

After observing the colocalization of NAGK, SNRPN, and DYNLRB1 proteins, we attempted to describe the role of NAGK and SNRPN in dynein-mediated cargo transport in SK-MEL-31 cells via observing the distribution pattern of melanosomes in the cell. We performed the NAGK, SNRPN, and DYNLRB1 protein overexpression and knockdown experiment on SK-MEL-31 cells to observe the distribution of melanosomes (Figure 8). Overexpressed and knockdown cells were treated with ciliobrevin D and nocodazole to disclose the involvement of NAGK, SNRPN, and DYNLRB1 proteins. ICC was performed with the melanosome-specific primary antibody (PMEL) on each condition. SK-MEL-31 cells were transfected with EGFP, NAGK, SNRPN, and DYNLRB1 overexpression plasmids, and the corresponding cells were treated with ciliobrevin D and nocodazole (Figure 8A). As compared to the control, EGFP-transfected cells (Figure 8A-a) and NAGK- (Figure 8A-b), SNRPN- (Figure 8A-c), and DYNLRB1- (Figure 8A-d) overexpressed cells revealed more centralized melanosome accumulation. The punctate accumulation of melanosomes was disrupted during the nocodazole treatment in the overexpressed plasmids (Figure 8A).

Furthermore, knockdown of the NAGK, SNRPN, and DYNLRB1 plasmids along with the control was performed in SK-MEL-31 cells (Figure 8B). In the control knockdown experiment, the melanosomes appeared punctate in the PMEL ICC during the ciliobrevin D treatment and distributed during the nocodazole treatment (Figure 8B-a). Similarly, the NAGK (Figure 8B-b), SNRPN (Figure 8B-c), and DYNLRB1 (Figure 8B-d) protein knockdown delayed the microtubule transport of melanosome. The melanosomes accumulated at the cell periphery during the ciliobrevin D treatment, which is the reverse outcome (central accumulation) observed in the overexpression experiment. Nocodazole treatment dispersed the melanosomes throughout the cell.

### 2.10. Neurons Derived from SNRPN-Depleted PWS Patient iPSC Lack Dendritic Development and Axonal Branching

We were curious to observe the effect of SNRPN depletion on neurodevelopment. To observe the effect of SNRPN depletion on neurodevelopment, we obtained patient- derived iPSC (PWS-2-9) with SNRPN deletion and normal human iPSC (UCH-HF-YK-27). We differentiated the human fibroblast-derived iPSC into neurons until day 30 in vitro. The morphology of the neurons was observed after fixing the neurons at day 30 of neuronal differentiation (Figure S5). The number of dendrites was higher on the normal human-iPSC-derived neuron, whereas the PWS-2-9-cell-derived neuron revealed decreased dendritic outgrowth from the cell body of the neuron. The branching of the dendrites and axon was also reduced in the disease model (Figure S5A). We also verified SNRPN depletion in the patient-derived (PWS-2-9) neuron vs. the normal cell-derived neuron via NAGK–SNRPN PLA. Very few NAGK–SNRPN PLA dots were observed in the PWS-2-9 disease model neurons as compared to the normal control neurons (Figure S5B), which confirmed the absence of SNRPN protein in the disease model cell line.

### 2.11. Exogenous NAGK and SNRPN Promote the Dendritic Development of PWS-2-9 iPSC- Derived Neurons

**Figure 9:**
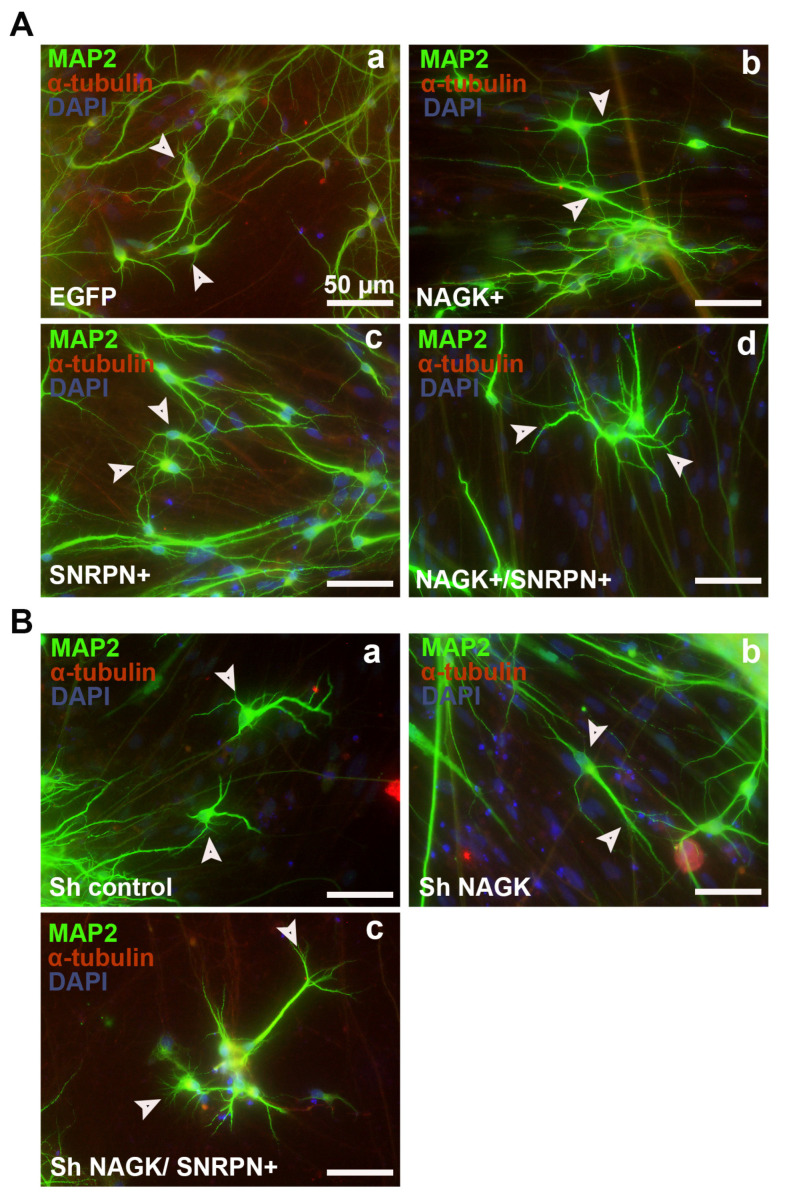
Overexpression and knockdown of NAGK and SNRPN in PWS 2-9 iPSC derived neuron. (**A**-**a**) ICC showing the overexpression effect of EGFP, (**A**-**b**) NAGK, (**A**-**c**) SNRPN, and (**A**-**d**) NAGK + SNRPN on the PWS-2-9-derived neurons observed at day 30 of neuronal differentiation. MAP2 (green) dendritic marker, α-tubulin (red), and cell body (DAPI). (**B**) ICC showing the knockdown effect of (**B**-**a**) Sh control, (**B**-**b**) Sh NAGK, and (**B**-**c**) Sh NAGK with SNRPN overexpression in the PWS-2-9-derived neurons at day 30 of neuronal differentiation. MAP2 (green) dendritic marker, α-tubulin (red), and cell body (DAPI). Scale bar of 20 µm applies to all the images. Arrowheads showed the cell body and process of the iPSC-derived neuron.

After observing the SNRPN depletion effect in human neurons, we decided to observe the effect of exogenous NAGK and SNRPN on the human-iPSC-derived neuron. The PWS-2-9-cell-derived iPSC neurons were overexpressed with the NAGK and SNRPN transgenes. ICC was performed with MAP2 (dendrite-specific marker, green) and α-tubulin (red) staining, and DAPI (blue) was used to stain the cell body of the neuron (Figure 9). The overexpression of EGFP, NAGK, SNRPN and the co-transfection of NAGK and SNRPN were performed. The EGFP-overexpressed neurons had limited dendritic processes (Figure 9A-a). The NAGK-transgene-overexpressed neurons showed increased dendritic numbers and branching from the cell body of the neuron (Figure 9A-b). Interestingly, during SNRPN overexpression, the number of dendrites and the frequency of branching increased. A similar effect was noticed when we overexpressed both NAGK and SNRPN (Figure 9A-d). During the knockdown using the scrambled gene, the dendritic number and branching (Figure 9B-a) were similar to the EGFP-transfected neuron. Similarly, the knockdown of NAGK also revealed reduced dendritic branching (Figure 9B-b). The knockdown of NAGK and overexpression of SNRPN induced an increment in dendritic protrusion from the cell body of the neuron, but the dendritic process lengths were reduced (Figure 9B-c). These findings reveal the crucial role of NAGK and SNRPN in axodendritic branching during neurodevelopment.

## 3. Discussion

The non-canonical role of NAGK in axodendritic development was reported previously [6,7,11]. Also, NAGK and SNRPN interaction in the neuron was confirmed using a yeast two-hybrid assay and PLA in our previous report [33]. However, a detailed study on the role of NAGK and SNRPN in neurodevelopment was not conducted. In this study, we explored the involvement of NAGK and SNRPN in neurodevelopment via overexpression and knockdown experiments. We observed the elevated expression of both NAGK and SNRPN at crucial phases of neurodevelopment (DIV/P 3 and 16) (Figure 1). We strengthen our PLA finding through an in vitro binding assay exhibiting the direct interaction between NAGK and SNRPN (Figure 2). These novel findings provided a clue that NAGK and SNRPN work together in neurodevelopment.

Defects in the SNRPN locus are pointed to as the crucial factor in the PWS phenotype [34,35,36,37,38]. Moreover, SNRPN’s role in the modulation of alternative splicing is widely accepted [39]. In addition, SNRPN’s involvement in cortical and spine development was also identified [12]. Despite these reports, the role of SNRPN in neurodevelopment is yet to be understood. Based on the previous reports, we hypothesized that NAGK and SNRPN may work together in neurodevelopment. In this study, the overexpression and knockdown of the NAGK and SNRPN proteins drastically modulated axodendritic development in the neuron (Figure 3 and Figure 4). Our finding strengthened the previous reports on the neurodevelopmental role of NAGK. Despite these findings, we still could not figure out the distribution variability of the NAGK–SNRPN complex in the neuron.

The interaction of NAGK with dynein motor protein DYNLRB1 was verified through different molecular biological analyses including PLA, yeast two-hybrid assay, and immunoprecipitation assays [16]. Studies on SNRPN’s interaction with DYNLRB1 are rare. One of the studies disclosed the interaction of the SmB protein with dynein cytoplasmic 1 heavy chain 1 (DYNC1H1) in neural trafficking [17]. However, the present study is the first report on the interaction of SNRPN and DYNLRB1 proteins in neurons. The colocalization of NAGK and DYNLRB1 proteins was reported at the somatodendritic areas and axonal growth cones [6]. Furthermore, NAGK and DYNLRB1 interaction was also involved during cell division [33]. Based on the previous findings, the role of NAGK and DYNLRB1 interaction is crucial for neurodevelopment.

In this study, we introduce the SNRPN protein as a new interacting partner with NAGK and DYNLRB1 proteins and regulate the retrograde cargo transport in the neuron. SNRPN’s gene locus is located within the paternally imprinted region of the genome that is critical in Prader–Willi syndrome (PWS). However, little is known about its behavior, except for its incorporation into snRNPs [40]. The SNRPN protein’s interaction with the essential neural protein neurochondrin (NCDN) and survival motor neuron (SMN) protein was supposed to have a role in protein localization, cell polarity, and trafficking of vesicles [41]. This study is the first report on DYNLRB1’s interaction with the SNRPN protein. The interaction of NAGK–DYNLRB1 and SNRPN–DYNLRB1 proteins was significantly upregulated in similar stages of neurodevelopment. Since the handling of neurons to observe the molecular mechanism is tedious, we observed the interaction of NAGK, SNRPN, and DYNLRB1 in the SK-MEL-31 cell line model using the dynein inhibitor ciliobrevin D [42,43,44] and microtubule-disrupting agent nocodazole [45,46]. The cells treated with ciliobrevin D exhibited the aggregation of melanosomes and the NAGK, SNRPN, and DYNLRB1 proteins. However, the dynein motors move the melanosomes toward the cell center along the microtubule [22]. The overexpression and knockdown of NAGK, SNRPN, and DYNLRB1 increased melanosome levels near the cell center, whereas knockdown of NAGK, SNRPN, and DYNLRB1 increased melanosome aggregation towards the cell periphery (Figure 8). These findings strongly suggest that NAGK, SNRPN, and DYNLRB1 are involved in microtubule transport in the neuron.

Studying neurological diseases is challenging due to limited access to brain material. However, generating induced pluripotent stem cells (iPSCs) from PWS patients can help overcome this obstacle [47,48,49,50,51]. We had chosen the SNRPN deletion cell derived from a PWS patient [52]. Studies have reported about the neuronal differentiation problem in PWS-derived iPSCs, but they could not elucidate the structure of PWS-derived neurons [51,52]. In the current study, we could differentiate the neuron stem cell to a neuron in 4 weeks and studied the morphology. To the best of our knowledge, very rare studies are reported on the transgene effect on the recovery from genetic neurodegenerative disease. NAGK and SNRPN’s introduction in the PWS-patient-derived neuron increased the number of dendrites and branching in the neuron. Also, the knockdown of the NAGK protein does not help to recover the SNRPN deletion effect in SNRPN cells. The overexpression of the SNRPN transgene increased the number of dendrites and branching in the neurons.

Dynein-mediated transport of NAGK and SNRPN may play a critical role in neuronal development, and the disruption of this transport can have deleterious effects on the developing brain. Moreover, it is unclear whether the loss of function of these genes affects other aspects of neuronal development and function beyond neurite outgrowth. Therefore, further research is needed to elucidate the role of SNRPN and NAGK in PWS and their potential as therapeutic targets for the disorder. Also, the precise mechanisms through which SNRPN and NAGK contribute to the neurological symptoms of PWS remain unclear. In vivo studies of the NAGK and SNRPN knock-in and knock-out mice model can help to strengthen the current findings, which could provide a fascinating future perspective.

## 4. Materials and Methods

### 4.1. Antibodies

For this study, the following antibodies were used in the experiments at the specified dilutions: mouse monoclonal NAGK (1:50 for PLA, 1:200 for ICC; Santa Cruz Biotechnology, Delaware Ave, CA, USA); chicken polyclonal NAGK (1:1000 for WB; GW22347, Sigma-Aldrich, Saint Louis, MO, USA); rabbit polyclonal NAGK (1:200 for ICC; 1:1000 for WB, ABclonal; A9070; Woburn, MA, USA); rabbit polyclonal DYNLRB1 (1:200 for ICC and 1:50 for PLA; ABclonal; A15197, Woburn, MA, USA); rabbit polyclonal SNRPN (1:50 for PLA and 1:200 for ICC, Proteintech Group; 11070-1-AP; Rosemont, IL, USA); mouse polyclonal SNRPN (1:200 for PLA and 1:500 for ICC; Abnova; 89-006-675; Walnut, CA, USA); mouse monoclonal His (1:100 for IP; ABclonal, Woburn, MA, USA); mouse monoclonal alpha-tubulin (1:25; 12G10; Developmental Studies Hybridoma Bank, University of Iowa, Iowa City, IA, USA); mouse monoclonal GFP (1:2000 for ICC; Invitrogen; A6455; Grand Island, NY, USA); chicken polyclonal MAP2 (1:5000 for ICC; Invitrogen; PA1-10005; USA); mouse monoclonal GST (1:1000 for WB; Invitrogen; MA4-004; Grand Island, NY, USA); rabbit monoclonal glyceraldehyde 3-phosphate dehydrogenase (GAPDH) (1:1000 for WB; 14C10; Cell Signaling technology; Danvers, MA, USA); rabbit polyclonal Actin (1:1000 for WB; Sigma-Aldrich; A-2066, Saint Louis, MO, USA). To detect the primary antibodies, Alexa Fluor 488 and 568 (Invitrogen, Grand Island, NY, USA) secondary antibodies were employed for fluorescent labeling.

### 4.2. Constructs and Plasmids

HEK293T cells were transfected with His-tagged pENTER-CMV-NAGK (CH817585) and pENTER-CMV-DYNLRB1 (CH803465) plasmids from Vigene Biosciences, Hollister, CA, USA, as well as the Myc-DDK-SNRPN (RR201341) and Myc-DDK-DYNLRB1 plasmids (RR210555) from Origene Technologies, Rockville, MD, USA. The NAGK gene was cloned on the pGSTag plasmid (21,877) from Addgene, Watertown, MA, USA, and pET-32a+ (69015-3) from EMD Biosciences, San Diego, CA, USA. The SNRPN gene from Myc-DDK-SNRPN plasmid was cloned using restriction digestion (BamHI-NotI) on the pET-32a+ vector for bacterial expression and pull-down assay. The NAGK and SNRPN overexpression and knockdown genes were cloned on the Adeno-associated virus (AAV) vector pAAV.CMV.PI.EGFP.WPRE.bGH (105,530). Cloned NAGK and SNRPN transgenes were packaged using the pAAV2/5 (104,964) and pAdDeltaF6 (112,867) plasmids [53,54,55,56,57,58]. The primary hippocampal neurons were transduced using AAV particles. ShRNA NAGK (TRCN0000037619), shRNA SNRPN (TRCN0000417615), and shRNA DYNLRB1 (TRCN0000153028) were cloned into the pLKO.1-puro vector (SHC002) from Sigma-Aldrich, Saint Louis, MO, USA. The AAV knockdown vectors were constructed via PCR amplification and restriction digestion cloning of the transgene from the pLKO.1-puro vector into the AAV.EGFP vector (105,530). PCR amplification was performed using the forward primer 5′-3′ (GATGGTGCTAGCTTCGCAAAACCAGCAAGAAAAGAAT) and reverse primer 5′-3′ (CATGCTGGATCCTAATTGTGGATGAATACTGCCATTT) and cloned with restriction digestion at the NheI and BamHI restriction sites of the AAV.EGFP vector.

### 4.3. Primary Neuronal Culture

Pregnant Sprague Dawley rats were purchased from KOATECH, Pyeongtaek, Republic of Korea, and raised in a light/dark cycle of 12/12 h with access to food and water ad libitum. On the 19th day of pregnancy, the pregnant rat was euthanized with isofluorane and the hippocampus of the fetus’s brain was collected for primary neuronal culture as previously described [59,60]. The hippocampal cells were seeded plated onto 12 mm-diameter poly-D-lysine-coated glass coverslips at a density of approximately 150 neurons/mm^2^ in a serum-free neurobasal media supplemented with B27, glutamate, and β-mercaptoethanol preincubated wells at 37 °C under 5% CO_2_ and 95% air. All experiments were conducted in accordance with the guidelines and approval from the Animal Care and Use Committee of Dongguk University, College of Medicine.

### 4.4. Cell Line and Transfection

HEK293T and SK-MEL-31 cells were acquired from the Korean Cell Line Bank located in Seoul, Republic of Korea. The culture media was DMEM and MEM (Invitrogen, Grand Island, NY, USA) with 10% fetal bovine serum and 1% penicillin-streptomycin (P-S). HEK293T cells were transfected with the desired plasmid for NAGK, SNRPN, and DYNLRB1 overexpression used in the in vitro binding assays. DH5α and NEB *E. coli* competent cells were used for plasmid production. Bacterial pure protein was expressed in BL-21 competent cells obtained from New England Biolabs, Rowley, MA, USA.

### 4.5. Patient-Derived iPSC Culture and Neuronal Differentiation

Induced pluripotent stem cells (iPSCs) of a PWS patient (PWS 2-9) and control UCH-HF-YK-27 cell lines derived from fibroblast were purchased from PWS iPSC Biobank University of Connecticut (UConn Stem Cell Core), Farmington, CT, USA. The fibroblast cells were induced into the stem cell using the lentivirus reprogramming method [61]. The PWS 2-9 cell contains the deletion of the paternal allele of the SNRPN gene on chromosome 15q11-q13 region [52]. Several protocols were collected and modified to maintain and differentiate the iPSCs into neural stem cells and mature neurons [29,48,49,50,51,61,62]. The iPSC lines were maintained in the feeder-free medium in the matrigel or vitronectin-coated culture vessels (mTeSR-1 or Essential 8 medium with 100 × P-S) with daily complete medium change. Differentiation into the neuronal stem cell was performed via the neural induction medium (neurobasal medium, neural induction supplement, and 100 × P-S) for 7 days. RevitaCell™ Supplement with Rho kinase inhibitor (ROCKi) was supplemented during the release and passage for 24 h. Neural stem cell expansion (for 4–6 days) was performed in the neural expansion medium (Neurobasal medium, Advanced DMEM/F-12, Neural induction supplement, and 100 × P-S) using geltrex-coated culture vessels. Neuronal stem cells were sequentially differentiated into neurons using a neural differentiation medium and PDL-coated glass coverslip. At first, Stem Pro NSC SFM complete medium: Knockout-DMEM/F-12, GlutaMAX-I supplement [2 mM], bFGF [20 ng/mL], EGF [20 ng/mL], stem pro neural supplement [2%], and 100 × P-S was used. After 48 h, a whole medium replacement was performed using neurobasal medium plus, B-27 plus serum-free supplement, culture one supplement, glutaMAX-I supplement, 2-phosphate sesquimagnesium salt hydrate (200 mM), and 100 × P-S. For faster neuronal differentiation, dibutyryl cAMP was added to the culture to a final concentration of 0.5 mM at day 7 for 3 days. Half of the volume of the medium was changed with fresh medium every three days for 30 days to develop into mature neurons.

### 4.6. Immunocytochemistry

Fixation of primary hippocampal cultures was performed using a previously described sequential paraformaldehyde/methanol fixation protocol [63]. Immunostaining was performed after blocking the hippocampal cultures with a blocking solution followed by primary antibody (detailed in the Section 4.1) incubation at 4 °C overnight. After washing the primary antibody thrice (10 min each) in pre-block, Alexa-conjugated secondary antibody (Invitrogen) incubation was performed as indicated and the samples were mounted on slides using VECTASHIELD antifade mounting medium for nuclear staining from Vector laboratories, Newark, CA, USA.

### 4.7. Proximity Ligation Assay

The PLA was conducted utilizing a Duolink kit (Sigma Aldrich, Saint Louis, MO, USA) with slight modifications to the manufacturer’s instructions. Fixed cells were exposed to primary antibodies in a pre-blocking buffer (5% normal goat serum, 0.05% TritonX-100 in PBS, pH 7.4) overnight at 4 °C. After washing the cells three times with pre-blocking buffer for 10 min each at room temperature (RT), secondary antibodies conjugated with oligonucleotides (PLA probe anti-mouse MINUS and PLA probe anti-rabbit PLUS) were diluted in pre-blocking buffer, added to the cells, and incubated for 2 h at 37 °C in a humidity chamber. The remaining steps of the assay were carried out according to the manufacturer’s instructions. Briefly, cells were washed with the wash buffer provided in the Duolink kit, and then exposed to the ligation mixture and ligase for 30 min at 37 °C to allow for hybridization and ligation of the DNA oligonucleotides. Subsequently, an amplification solution containing polymerase was added, resulting in a rolling circle amplification reaction. The amplified product was detected using complementary fluorescently labeled oligonucleotides. In the case of combining PLAs with ICC, the PLA reactions were performed first, followed by overnight incubation of primary antibodies with the cells at 4 °C, and then incubation with fluorophore-conjugated secondary antibodies according to the ICC procedure.

### 4.8. Pull-Down and Co-Immunoprecipitation Assays

For the pull-down assay, His-tagged NAGK, Myc-DDK-SNRPN, His-DYNLRB1, and Myc-DDK-DYNLRB1-tagged plasmids were transiently transfected into HEK293T cells using calcium-phosphate-mediated transfection. After 48 h of incubation, the cells were lysed and collected using Pierce™ IP Lysis Buffer (87,787; ThermoFischer scientific, Rockford, IL, USA) (25 mM Tris-HCl pH 7.4, 150 mM NaCl, 1 mM EDTA, 1% NP-40, and 5% glycerol; pH 7.4) containing protease inhibitor cocktail (Thermo Scientific, Rockford, IL, USA), followed by washing with cold PBS. The supernatant was then allowed to bind to MagListo™ Ni-NTA magnetic silica resin (Bioneer, Daejeon, Korea) as per the manufacturer’s instructions for the pull-down assay.

The co-immunoprecipitation assay was performed with a His-tagged antibody using puredown Protein G-Agarose beads (P9202-015; genDEPOT; Baker, TX, USA). The beads (40 µL) were incubated with the individual and co-transfected protein lysate (500 µg) of His-NAGK and Myc-DDK-SNRPN plasmids. The beads were incubated overnight with His-antibody and control IgG at 4 °C with rotation. After washing the beads four times in 100 mM phosphate-buffered saline (PBS), the beads were boiled in 50 µL of 2 × lamelli buffer (65.8 mM Tris-HCl, pH 6.8, 2.1% SDS, 26.3% (*w*/*v*) glycerol, 0.01% bromophenol blue) and the elution was collected for further analysis.

### 4.9. Western Blot

The whole brain of the mouse pup and primary hippocampal culture were washed with ice-cold PBS and homogenized on ice for 30 min using 1 × RIPA buffer containing 1% protease inhibitor. The mixture was then centrifuged at a speed of 13,000 rpm for 15 min, and the resulting supernatant was collected. Protein concentrations were determined using the bicinchoninic acid (BCA) method. The protein sample of each group was separated using SDS-PAGE, transferred to 0.2 μm polyvinylidene difluoride (PVDF) membranes, and blocked in 5% skimmed milk for 1.5 h. Incubation of the membranes was performed with the desired primary antibodies overnight at 4 °C, followed by incubation with goat anti-mouse/rabbit/chicken HRP secondary antibodies for 1 h at room temperature. The PVDF membranes were soaked in ECL luminous solution and detected using the Tanon 5200 Luminescence imaging system. Images were analyzed using Image J software version 1.43. Each band was compared with the GAPDH band (loading control) in the study.

### 4.10. Image Acquisition and Analysis

The phase contrast microscopy images were acquired using a Leica DM IL LED microscope from Gyeongbuk Technopark Advanced Medical Convergence Textile Center, Daegu, South Korea, which was equipped with a Leica DFC3000 G digital monochrome camera (1296 × 966 pixels) featuring EX view HAD technology and controlled by Leica LAS X software (Leica Microsystems ver. 2.0.2.15022, Wetzlar, Germany) (20×, NA 0.4). Immunocytochemistry images were captured with an Olympus BX53^®^ polarizing light microscope equipped with an Olympus DP72^®^ camera, controlled by cellSens™ software (Olympus Entry. Ink 2020, Version 1.0) (20×, NA 0.5 and 40×, NA 0.75) (Center Valley, PA, USA).

Image analysis for morphometry and quantitative analysis was performed using Image J software (version 1.49) with the simple neurite tracer plug-in (National Institute of Health, Bethesda, MD, USA), concentric circle plug-in, and Sholl plug-in (http://biology.ucsd.edu/labs/ghosh/software (accessed on 12 March 2023)), as previously described. The neurite area for the treated and control neurons was evaluated using the Neurphology J plug-in with Image J software (version 1.43).

### 4.11. Statistics

The data are represented as the mean ± standard error of the mean (SEM) from the three independent experiments unless otherwise specified. Statistical analysis was conducted using GraphPad Prism version 8.0.0 for Windows (San Diego, CA, USA). The statistical significance was assessed using different tests, including Student’s t-test and one-way and two-way ANOVA, followed by multiple comparison tests such as Dunnett’s and Tukey, and statistical significance was defined as *p* values ≤ 0.05.

## 5. Conclusions

In conclusion, our study demonstrated a significant upregulation of NAGK and SNRPN interaction in the cell body and processes of neurons during the neurite outgrowth and axodendritic development stages of neurodevelopment. Additionally, we successfully confirmed the microtubule-mediated transport of NAGK–SNRPN through their interaction with DYNLRB1 in both neurons and the SK-MEL-31 cell line. Remarkably, we found that NAGK–SNRPN interaction rescued the compromised axodendritic outgrowth observed in the SNRPN deletion model of PWS-2-9-patient-derived iPSC neurons. These findings have important implications for developing therapeutic approaches for neurodegenerative diseases.

## Data Availability

The data will be provided upon reasonable request.

## Supplementary Materials

The following supporting information can be downloaded at: https://www.mdpi.com/article/10.3390/ijms241411672/s1.
