# Supplementary material for: N-Acetylglucosamine Kinase–Small Nuclear Ribonucleoprotein Polypeptide N Interaction Promotes Axodendritic Branching in Neurons via Dynein-Mediated Microtubule Transport
Source: Int J Mol Sci. 2023 Jul 19;24(14):11672. doi: 10.3390/ijms241411672 (PMC10380243; doi:10.3390/ijms241411672)
Supplement: Supplementary file 1 [file ijms-24-11672-s001.zip › Supplementary information.pdf]

Supplementary material

Article

NAGK-SNRPN interaction promotes axodendritic branching in neurons via dynein-mediated microtubule transport

Binod Timalsina [1], Ho Jin Choi [1] and Il Soo Moon [1,*]

[1] Department of Anatomy, Dongguk University College of Medicine, Gyeongju 38066, Republic of Korea

* Correspondence: moonis@dongguk.ac.kr (I.S.M).

Supplementary Figures

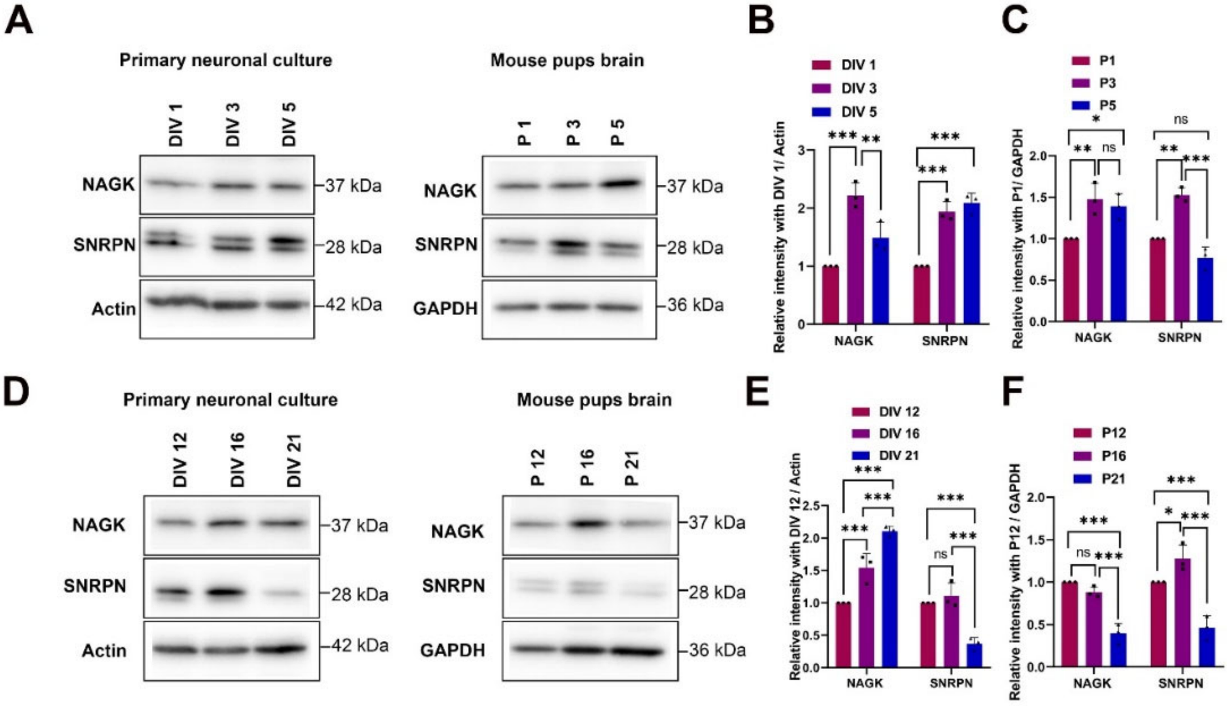

Figure S1. Normal expression of NAGK and SNRPN in primary neurons and mouse whole brain lysate. (**A**) Western blot showing the normal expression of NAGK and SNRPN at (DIV and P) 1/ 3/ 5. (**B**) Bar graph showing the intensity measurement of NAGK and SNRPN in primary neuron using actin as the loading control at DIV 1/ 3/ 5. (**C**) Bar graph showing the intensity measurement of NAGK and SNRPN from mouse pups whole brain lysate using GAPDH as the loading control at P1/ 3/ 5. (**D**)Western blot showing the normal expression of NAGK and SNRPN at (DIV and P) 12/ 16/ 21. (**E**) Bar graph showing the intensity measurement of NAGK and SNRPN in primary neuron using actin as the loading control at DIV 12/ 16/ 21. (**F**) Bar graph showing the intensity measurement of NAGK and SNRPN from mouse pups whole brain lysate using GAPDH as the loading control at P12/ 16/ 21. Bars represent the mean ± SD (n = 3) for western blot analysis. Statistical significance was compared by one- way ANOVA with Dunnett's multiple comparisons test: *$P < 0.05$, **$P < 0.01$, and ***$P < 0.001$.

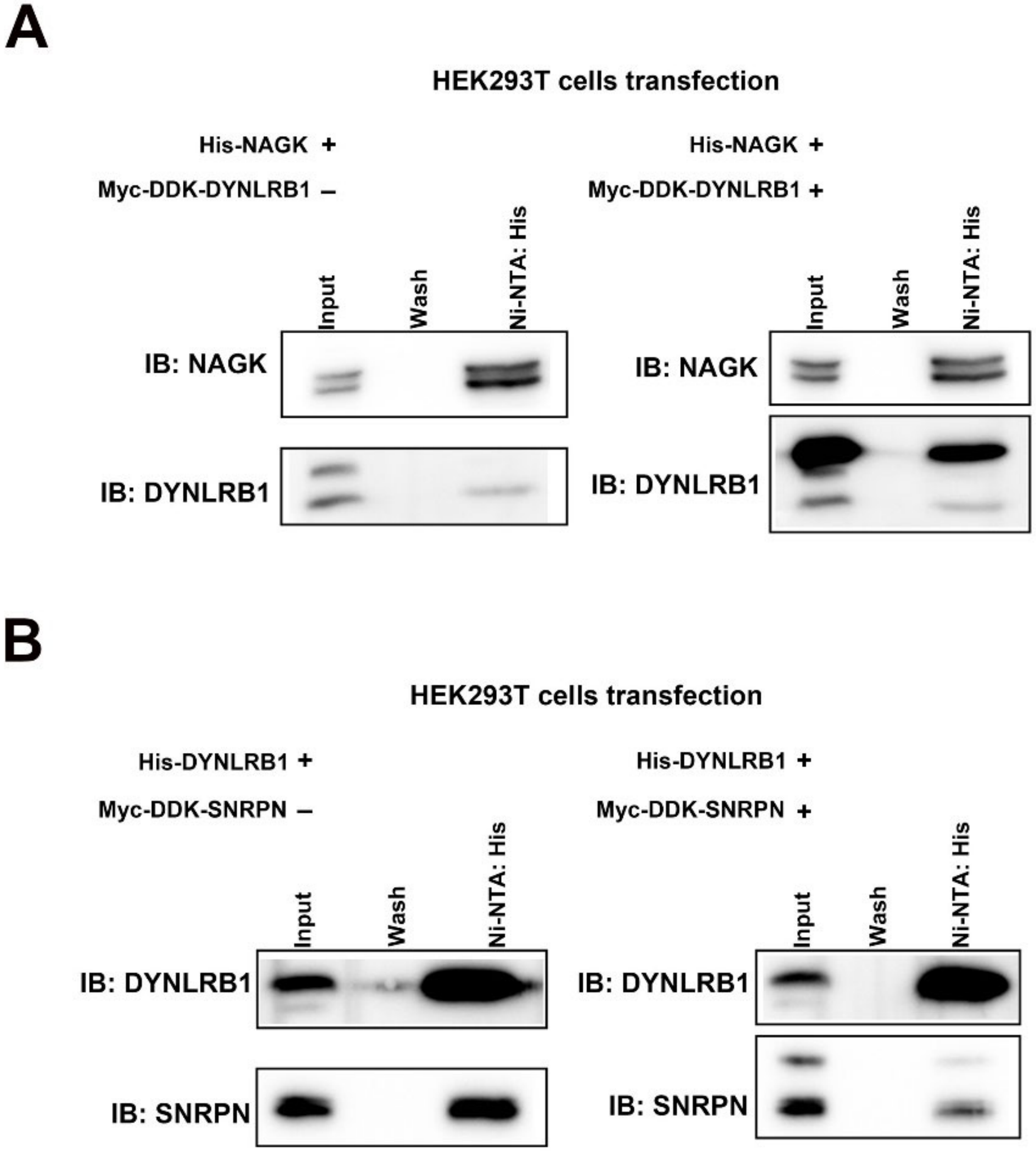

Figure S2. Pull down assays showing the NAGK, SNRPN, and DYNLRB1 interactions. (**A**) His pull down assay showing the interaction with His-NAGK and Myc-DDK-DYNLRB1 in HEK293T cell lysate. (**B**) His pull down assay showing the interaction with His-DYNLRB1 and Myc-DDK-SNRPN in HEK293T cell lysate.

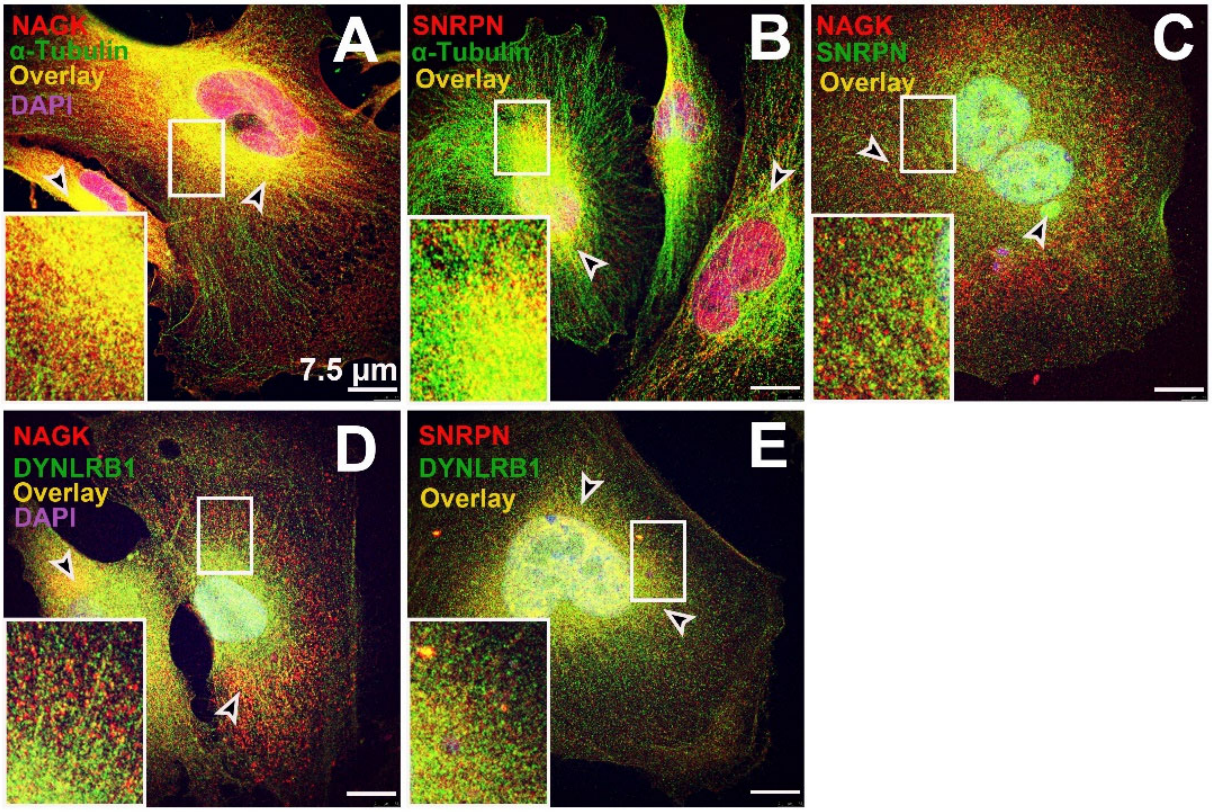

Figure S3. Immunocytochemistry showing NAGK, SNRPN, and DYNLRB1 colocalization on microtubule in SK-MEL-31 cells. (**A**) ICC image of NAGK (red), α-tubulin (green), colocalization (overlay), and DAPI as a nuclear stain. (**B**) SNRPN (red), α-tubulin (green), colocalization (overlay), and DAPI as a nuclear stain. (**C**) NAGK (red), SNRPN (green), and colocalization (overlay), and DAPI as a nuclear stain. (**D**) NAGK (red), DYNLRB1 (green), colocalization (overlay), and DAPI as a nuclear stain. (**E**) SNRPN (red), DYNLRB1 (green), colocalization (overlay), and DAPI as a nuclear stain. Scale bar,7.5 μm applies to all the images.

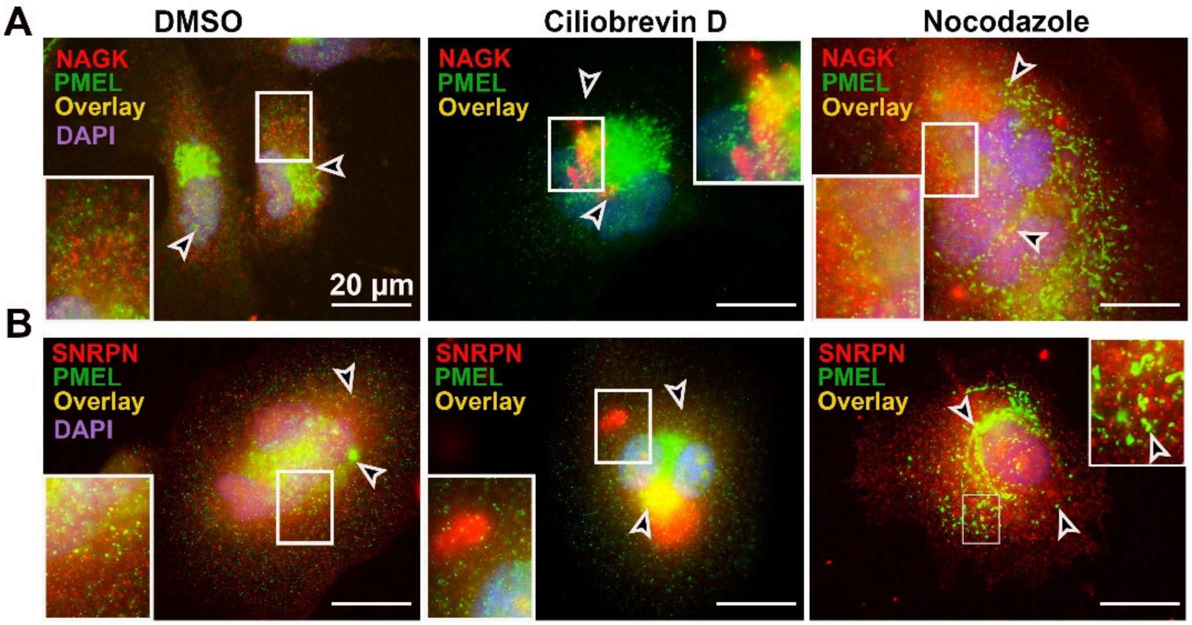

Figure S4. Immunocytochemistry showing NAGK and SNRPN colocalization of melanosome in SK-MEL-31 cell line. ICC showing the treatments with (DMSO, ciliobrevin D, and nocodazole). (**A**) NAGK (red), PMEL (green), and colocalization (overlay). (**B**) SNRPN (red), PMEL (green), and colocalization (overlay). Scale bar, 20 μm and inset, 6.66 μm applies to all the images.

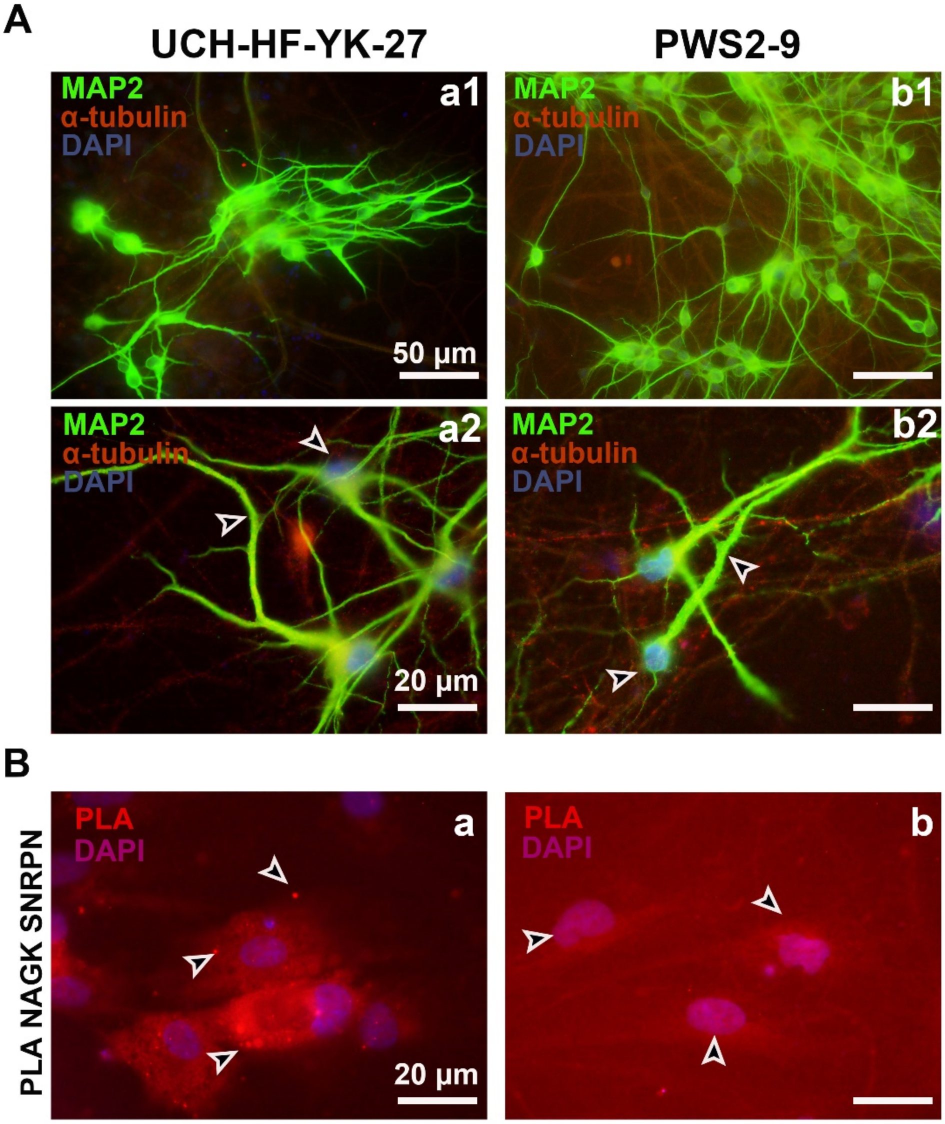

Figure S5. Neuronal morphology and proximity ligation assay of the normal and PWS patient derived iPSC. (**A**) ICC showing the dendrites (MAP2, green) and whole process of the neuron (α-tubulin, red) and the cell body (DAPI) at Day 30 of neuronal differentiation. (**A-a1**) The morphology of the UCH-HF-YK-27 cells in the low and (**A-a2**) high magnification. (**A-b1**) PWS 2-9 iPSC derived neurons in the low and (**A-b2**) high magnification. (**B**) PLA showing the NAGK-SNRPN dots (red) and the cell body of neuron (DAPI). (**B-a**) NAGK-SNRPN PLA in the normal UCH-HF-YK-27 cells. (**B-b**) NAGK-SNRPN PLA in the diseased PWS 2-9 cells. Scale bar, 50 μm (**A-a1** and **A-b1**) and 20 μm applies to all the images.
